# Polycomb group-mediated histone H2A monoubiquitination in epigenome regulation and nuclear processes

**DOI:** 10.1038/s41467-020-19722-9

**Published:** 2020-11-23

**Authors:** Haithem Barbour, Salima Daou, Michael Hendzel, El Bachir Affar

**Affiliations:** 1 Maisonneuve-Rosemont Hospital Research Center and Department of Medicine, University of Montréal, Montréal, H3C 3J7 QC Canada; 2 Lunenfeld-Tanenbaum Research Institute, Sinai Health System, Toronto, ON M5G 1X5 Canada; 3 Departments of Oncology and Cell Biology, University of Alberta, Room 3332 Cross Cancer Institute, 11560 University Avenue NW, Edmonton, T6G 1Z2 AB Canada

**Keywords:** Biochemistry, Cancer, Molecular biology, Epigenetics

## Abstract

Histone posttranslational modifications are key regulators of chromatin-associated processes including gene expression, DNA replication and DNA repair. Monoubiquitinated histone H2A, H2Aub (K118 in Drosophila or K119 in vertebrates) is catalyzed by the Polycomb group (PcG) repressive complex 1 (PRC1) and reversed by the PcG-repressive deubiquitinase (PR-DUB)/BAP1 complex. Here we critically assess the current knowledge regarding H2Aub deposition and removal, its crosstalk with PcG repressive complex 2 (PRC2)-mediated histone H3K27 methylation, and the recent attempts toward discovering its readers and solving its enigmatic functions. We also discuss mounting evidence of the involvement of H2A ubiquitination in human pathologies including cancer, while highlighting some knowledge gaps that remain to be addressed.

## Abstract (web summary)

Histone H2A monoubiquitination on lysine 119 in vertebrate and lysine 118 in Drosophila (H2Aub) is an epigenomic mark usually associated with gene repression by Polycomb group factors. Here the authors review the current knowledge on the deposition and removal of H2Aub, its function in transcription and other DNA-associated processes as well as its relevance to human disease.

## Introduction

Eukaryotic DNA is organized into a highly ordered structure called chromatin that can be broadly classified as either euchromatin (relaxed form) or heterochromatin (compacted form). The building blocks of this structure are nucleosomes, formed by the association between chromosomal DNA and histone octamers (dimers of H2A-H2B and H3-H4) to form arrays of nucleosomes connected by a shorter DNA linker with variable lengths. The crystal structure of the nucleosome revealed an organization of the 146 base pair DNA fragment around the histone octamer, with the histone N-terminal and C-terminal tails extending away from the nucleosome core^1^. Importantly, the histone tails are targeted by several posttranslational modifications (PTMs), which, in addition to giving an epigenetic dimension to chromatin, are involved in the regulation of virtually all DNA-dependent processes. These PTMs are catalyzed by a wide spectrum of chromatin modifying factors, including Polycomb group (PcG) and Trithorax group (TrxG) complexes, that act as molecular machines to orchestrate chromatin-associated processes^2–6^. Indeed, many PTMs are found on specific conserved residues of histone tails, including phosphorylation, methylation, acetylation, and ubiquitination where they regulate DNA replication, DNA damage/repair, and gene transcription^2–6^. Histone PTMs play important roles in a wide spectrum of biological processes including cell proliferation, cell fate determination, and differentiation as well as stress responses. Many studies have associated distinct profiles of histone modifications with human pathologies and it is increasingly recognized that alterations of chromatin function are causally linked to disease development and progression^6–8^.

**Fig. 1:**
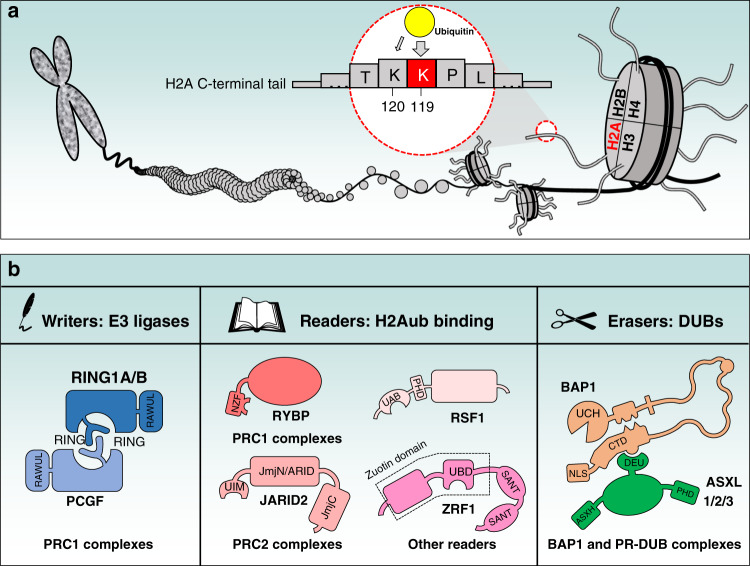
Mammalian H2AK119ub (H2Aub) regulation by writers (ubiquitin E3 ligases), readers (ubiquitin-binding proteins) and erasers (deubiquitinases). **a** Schematic representation of chromatin and the mammalian H2AK119 ubiquitination site by PRC1 E3 ligase. Note that the mammalian H2AK120 site is a minor site for PRC1-mediated ubiquitination. **b** Schematic representation of known modulators of H2Aub. Important functional domains are indicated. RING: Really Interesting New Gene, UBD: Ubiquitin-Binding Domain, UIM: Ubiquitin Interaction Motif, NZF: Nuclear protein localization 4 Zinc Finger, UAB: Ubiquitin-Associated Domain, RAWUL: Ring-finger and WD40 associated Ubiquitin-Like, PHD: Plant Homeodomain, UCH: Ubiquitin C-terminal Hydrolase, CTD: C-Terminal Domain, NLS: Nuclear Localization Signal, DEUBAD (DEU): DEUBiquitinase Activating Domain, ASXH: HARE-HTH of ASXLs.

Polycomb group (PcG)-mediated H2A ubiquitination occurs on K119 in vertebrates, K118 in Drosophila and K121 in Arabidopsis and will be hereafter referred to as H2Aub^9,10^. The function and genomic localization of H2Aub remained a mystery for a long time, but this situation has changed with the identification of its E3 ubiquitin ligase, the PRC1 complex, and its obligate components RING1A or RING1B. Indeed, inactivation of mammalian RING1A/RING1B or their Drosophila orthologue Sce completely abolishes H2A ubiquitination^11–19^. In this review, we will outline the functions of the complexes and factors that attach ubiquitin on H2A (writers), decode the ubiquitination signal (readers) or remove ubiquitin from this histone (erasers) (Fig. 1). We discuss our current understanding of the link between H2Aub and chromatin-associated processes and provide an update on the potential role of H2Aub deregulation in the development of human pathologies (additional definitions are listed in Box 1).

Box 1 Definitions CpG: DNA regions rich in CG nucleotides found in the genome and are often protected from cytosine methylation.

CUBI: Composite Ubiquitin-Binding Interface is a protein interaction interface formed by the catalytic domain of BAP1, its C-terminal domain and the DEUBAD of ASXLs.

DEUBAD: DEUBiquitinase ADaptor is a conserved domain found in Asx/ASXLs, RPN13 and INO80G and is responsible for regulating DUB activity.

EZH: Enhancer of Zeste Homolog 2 is the methyltransferase enzyme of the PRC2 complex that catalyzes histone H3K27 methylation and corresponds to E(z) in Drosophila. Mammalians have two paralogues of EZH (EZH1 and EZH2).

H3K36me3: A histone PTM found on the gene body of actively transcribed genes.

H3K4me1: A histone PTM found on active enhancers.

H3K4me3: A histone PTM found at gene promoters and is a landmark of activate genes.

Homeobox (Hox): Originally identified in Drosophila and correspond to a group of genes that are responsible for defining the identity of body segments along the anterior/posterior axis.

PBAF: Polybromo-associated BAF complex is a SWI/SNF family chromatin-remodeling complex that regulates transcription in ATP-dependent manner.

PHD: Plant homeodomain (PHD) is a small zinc finger domain with similarities to RING fingers. PHD can bind histone PTMs such as methylated lysine. Some PHD domains can also bind an unmodified lysine.

PTEN: Phosphatase and Tensin Homolog deleted on Chromosome 10 (PTEN) is a dual phosphatase that uses protein and lipid as substrates.

RING: Really Interesting New Gene domain is a distinct type of zinc finger domain found in E3 ubiquitin ligases.

TrxG: Transcriptional activators that counteract PcG action. TrxG can be histone-modifying complexes or ATP-dependent chromatin-remodeling complexes.

UCH: Ubiquitin C-terminal Hydrolase is the catalytic domain found in the UCH family of deubiquitinases that include BAP1.

## Mechanisms of H2Aub deposition

### Genome-wide distribution of H2Aub on chromatin

H2Aub is a highly conserved histone modification found in eukaryotic organisms including animals and plants. Although it is still unclear when this modification was established during the course of evolution, H2Aub seems to be present in certain unicellular eukaryotes including diatoms and ciliates^20,21^. In mammalian cells, it is estimated that H2A is the most ubiquitinated protein, with up to 10% of total H2A harboring this PTM^22^. Early studies relied on visualizing the global levels of H2Aub in mammalian cells indicated that this modification is enriched in heterochromatic X and Y chromosomes (XY bodies) in spermatocytes and inactive X chromosome in female cells and was thus linked to gene silencing^11,23–25^. ChIP-seq studies in Drosophila cell lines revealed that H2Aub is broadly distributed on the genome, and is found in transcription start sites (TSS), gene body, and intergenic regions^26^. Moreover, H2Aub colocalizes with H3K27me3, a transcription repressive mark catalyzed by the PcG PRC2 complex (section “Positioning PRC1 complexes within the PcG landscape”)^26^. However, H2Aub can also exist in chromatin regions independently of H3K27 methylation^26^. In the plant Arabidopsis, H2Aub marks are also widespread with a significant colocalization with H3K27me3 on repressed genes, although about half H2Aub signals are not associated with detectable H3K27me3^27^. ChIP-seq studies in mammalian cells also revealed that H2Aub is mostly associated with repressed genes^14–16,28–32^. For instance, in mouse embryonic stem cells (mESC), strong H2Aub signals are associated with promoters of repressed genes, notably PcG targets, which are often found within high-density CpG regions^15,16,31–34^. In addition, low levels of H2Aub are observed throughout the genome^32^. Importantly, a strong overlap between H2Aub and H3K27me3 is observed in stem cells and other cell types^14–16,28,29,31,32^. Of note, in several systems, low H2Aub signals could also be detected on active chromatin^14,27,29,31^. However, it is important to note that several factors can result in artificial H2Aub chromatin occupancy. These include the widespread nature of H2Aub, the chemical crosslinking conditions, and the stringency on ChIP-seq peak calling. Moreover, the presence of heterogeneous cell populations such as mixed differentiation states or asynchronous cell populations, can also complicate interpretation of H2Aub distribution. Thus, it is currently difficult to ascertain a definitive link between H2Aub and active chromatin.

In summary, the deposition of H2Aub is associated with repressed genes, notably PcG targets, and strongly overlaps with H3K27me3. H2Aub might also be associated with other chromatin contexts, possibly defining subcategories of H2Aub targets with specific histone PTMs and transcriptional states. Clearly, how the interplay between H2Aub and other histone PTMs orchestrates chromatin function is still not fully understood. Quantitative analysis of the genomic distribution of H2Aub in diverse cellular systems would shed light on how this PTM regulates chromatin-dependent processes in response to pathophysiological signaling.

### Positioning PRC1 complexes within the PcG landscape

The roots of PcG genes date back to early studies characterizing genes that regulate development and anterior/posterior body segments identity using the Drosophila model^2,5,35^. It was found that defects in the regulation of Homeobox (Hox) genes induces distinct morphological phenotypes characterized by their ectopic expression in other body segments, a phenomenon known as homeotic transformation^2,5,35^. We know now that the expression of Hox genes is regulated by two groups of genes, the PcG and the Trithorax group (TrxG), with opposing roles as repressors and activators, respectively^2,5,35^. Moreover, it is well established that the PcG protein family are epigenetic regulators that play critical roles in body patterning during development and in maintaining cellular identity^2,5,35^.

**Fig. 2:**
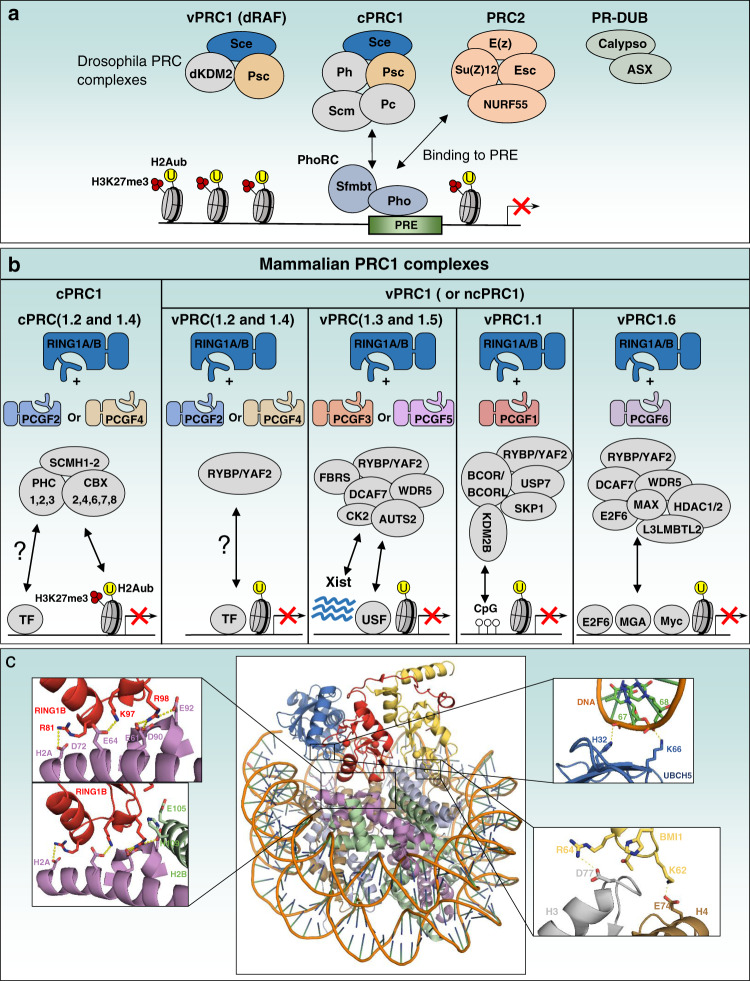
PcG complexes in Drosophila and PRC1 complexes in mammals. **a** Schematic representation of the five PcG complexes in Drosophila: canonical (cPRC1) and variant (vPRC1:dRAF) PRC1 complexes, PRC2 complex, PR-DUB complex and PhoRC complex. PhoRC binds to gene regulatory regions via the interaction of its subunit Pho with specific DNA sequences, PRE (PcG Responsive Elements). Pho can also directly bind to components of cPRC1 complexes to mediate their recruitment even in the absence of H3K27me3. The arrows indicate protein interactions responsible for the recruitment of PcG complexes to the PRE. **b** Schematic representation of the canonical (cPRC1) and variant (vPRC1) PRC1 complexes in mammalians. RING1A or RING1B are obligate components of the PRC1 complexes. Their association with either PCGF2/PCGF4 or other PCGFs (PCGF1, PCGF3, PCGF5, PCGF6) lead to the formation of cPRC1 or variants vPRC1 complexes, respectively. CBXs/HPHs and RYBP/YAF2 associate in mutually exclusive manner with RING1A/RING1B for the formation of cPRC1 and vPRC1 complexes, respectively. H3K27me3 and CpG islands recruit cPRC1 and vPRC1/PCGF1 complexes respectively. Transcription factors (TF), USF and other chromatin factors (E2F6, MGA, MYC) might also be involved in the recruitment of PRC1 to chromatin. The arrows indicate protein-protein or protein DNA/RNA interactions responsible for the recruitment of PRC1 complexes to chromatin. Ubiquitination is shown as the yellow circle and H3K27me3 is shown by the three red circles (**a**, **b**). **c** In the center, crystal structure of a minimal human PRC1 complex (RING1B /BMI1-UbcH5c) interacting with the nucleosome core particle (NCP) (PDB: 4R8P). The left panels show the contact between RING1B and H2A/H2B acidic patch. The top right panel shows the DNA-UBCH5 interactions. BMI1 also makes polar contacts with Histone H3 and H4 as shown in the bottom right panel. Interaction of RING1B with BMI1 on one hand and histone H2A on the other hand is essential for establishing H2Aub on the latter.

In Drosophila, PcG factors are divided into five multi-protein repressor complexes including the canonical Polycomb repressive complex 1 (cPRC1), dRing-associated factors (dRAF) complex (vPRC1), Polycomb repressive complex 2 (PRC2), Polycomb repressive DUB complex (PR-DUB) and Pho-repressive complex (PhoRC)^5,35–38^ (Fig. 2a). PRC1 and PRC2 are the most studied complexes and both have a wide range of variants. The PRC1 complex components are Polycomb (Pc), Sex Comb Extra (Sce or dRing), Posterior Sex Combs (Psc), Polyhomeotic (Ph) and Sex Comb on Midleg (Scm), whereas PRC2 core components are Enhancer of Zeste (E(z)), Suppressor of Zeste 12 (Su(Z)12), Extra Sex Combs (Esc) and Nucleosome Remodelling Factor 55 (Nurf55). PRC1 and PRC2 complexes ensure ubiquitination of H2AK118 and methylation of H3K27 through the E3 ubiquitin ligase Sce and the methyltransferase E(z), respectively^2,5,35^. dRAF contains Sce, Psc and the lysine demethylase dKDM2 and is also associated with H2Aub deposition^37^. PhoRC is composed by Pleiohomeotic (Pho) and the Scm-related protein containing four MBT domains (Sfmbt) protein which confer sequence-specific DNA recognition and chromatin binding^36,39^ (section “Deposition of H2Aub and coordination with H3K27me3”). Finally, the PR-DUB complex which deubiquitinates H2Aub contains the DUB Calypso and Additional Sex Comb Asx^38^. Asx and it mammalian orthologue ASX-Like 1 (ASXL1) possess dual roles of enhancing PcG and Trithorax functions, and belongs to a group of factors termed Enhancers of Trithorax and Polycomb (ETP)^40,41^ (section “Deubiquitination of H2Aub by PR-DUB and BAP1”). Overall, the diversity of Drosophila PcG complexes suggests that multiple mechanisms of gene repression can take place to ensure proper gene silencing during development and homeostasis.

The PcG complexes are much more diverse in mammals. Focusing on PRC1, recent studies provided a classification of canonical (cPRC1) and variant PRC1 (vPRC1) complexes depending on their subunit composition^13,14,32,42^ (Fig. 2b). The two Sce orthologues RING1A (RNF1) or RING1B (RNF2) are obligate and mutually exclusive components of PRC1 complexes and their depletion results in a complete loss of H2Aub chromatin signals^11–16^. The mammalian orthologues of Pc, Psc, and Ph are respectively the H3K27me3-binding chromobox family (CBX2/4/6/7/8), the PcG RING fingers family (PCGF1/2/3/4/5/6) and the human Ph family (PHC1/2/3) proteins^13,14,32,42^. Two other factors, RING1-Yin Yang1 (YY1) Binding Protein (RYBP) and YY1-Associated Factor 2 (YAF2), are also associated with a subset of PRC1 complexes in a mutually exclusive manner with CBX and HPH factors^13,14,32,42^. Thus, cPRC1 complexes are described as the association of RING1A or RING1B, CBXs, HPHs and either PCGF4 (BMI1) or PCGF2 (MEL18) and vPRC1 variants are composed of RING1A or RING1B, RYBP or YAF2 and one of the six PCGF factors^13,14,32,42–47^ (Fig. 2b). The wide diversity of PRC1 complexes in higher eukaryotes could be explained by a functional specialization that has been imposed during evolution to fine-tune gene silencing. Combinatorial interactions within PRC1 complexes and between PRC1 complexes and other factors would tailor repression mechanisms to specific cell fate decisions. It will be interesting to determine the dynamics of PRC1 complexes with a focus on transient interactions that take place at gene regulatory regions, in response to signaling events and cell fate transitions, and determine their relevance to H2Aub deposition and function.

### Deposition of H2Aub and coordination with H3K27me3

*Role of DNA-binding proteins in H2Aub deposition***:** Pioneering studies in Drosophila indicated that PcG-mediated repression relies on specific DNA sequences called PRE (or PcG Responsive Elements)^48,49^. PRE-mediated gene silencing was later found to involve DNA-binding PcG proteins such as pleiohomeotic Pho and Pho-like, whose vertebrate orthologue is YY1^36,50–53^. For instance, Pho can bind to components of the PRC1 complex and recruit its subunits directly to DNA, in the absence of nucleosomes^53,54^ (Fig. 2a). In addition, Sfmbt subunit of PhoRC can directly interact with PRC1 and promote its recruitment at PRE^55^. This might explain the presence of H2Aub in chromatin regions in the absence H3K27me3^26^. Thus, PhoRC is possibly the first PcG to be recruited to DNA and to nucleate repression^56^. Nonetheless, further work is needed to define the exact contribution of PhoRC complexes in coordinating H2Aub genomic distribution during Drosophila development and how Pho and other transcription factors orchestrate the function of PcG complexes to ensure gene silencing. The dRAF complex contains dKDM2 which possesses a CXXC-type zinc finger capable of direct DNA binding^57^. dRAF was shown to promote both ubiquitination of histone H2AK118 and demethylation of histone H3K36me2^37^. However, the exact role of DNA binding by dKDM2 in H2Aub deposition has not been established. In addition, dKDM2 mutant flies are viable^58,59^, arguing against a major role of this factor in H2Aub deposition and PcG repression.

Studies in high-order organisms failed to define true PREs, suggesting divergent modes of H2Aub deposition and regulation^35^. Recent biochemical and genome-wide studies indicated that PRC1 complex composition and DNA-binding factors are important in defining its recruitment and H2A ubiquitination^13–16,32,42,45,60^ (Fig. 2b). For instance, PCGF factors exert specific and overlapping roles in the establishment of H2Aub and H3K27me3^13,14,32^. In particular, recruitment of vPRC1 complexes harboring KDM2B, which binds DNA on unmethylated CpGs, through its CXXC zinc finger domain, plays an important role in H2A ubiquitination^13,33,61–63^. Several transcription factors can also be involved in the recruitment of canonical or vPRC1 complexes to chromatin, including REST, USF1/2, MGA, and E2F6^14,64–69^ (Fig. 2b). It is important to note that the exact contribution of many transcription factors to the deposition of H2Aub at specific target genes remains incompletely defined and further studies are needed to establish how transcription factors orchestrate H2Aub deposition depending on cell type and chromatin contexts.

*Impact of H3K27me3 on H2Aub deposition*: An initial model involved PRC2 recruitment at PcG target genes and recognition of H3K27me3 by Pc through its trimethyl-lysine binding domain known as chromodomain^49,53,70–73^. While this implied that PRC2 and PRC1 are sequentially recruited to the same regions to deposit H3K27me3 and subsequently H2Aub, respectively, many studies provided evidence for different mechanisms of H2Aub deposition^53–55^. First, as mentioned above, in Drosophila, PRC1 can be directly recruited to chromatin through PhoRC^55^. Second, mutation of Drosophila E(z), which strongly reduces H3K27me3, had only a minimal effect on the global levels of H2Aub^26^. Interestingly, while a significant reduction of H2Aub was observed around PREs following inactivation of PRC2, only a marginal decrease of global H2Aub levels was observed^74^. In mammals, several studies indicated that the bulk of genomic H2Aub does not depend on PRC2 and H3K27me3^45,75–78^. For example, in mESC which lacks the PRC2 component EED, global H2Aub levels remain unchanged in the absence of H3K27me3^45,75^. Moreover, it was found that deposition of H2Aub on inactivated X chromosome is independent of PRC2 and precedes H3K27me3 deposition^79,80^. However, other studies in mESC and somatic cells showed that inactivation of PRC2 can result in significantly decreased levels H2Aub both globally and on gene regulatory regions^81,82^. The reasons behind these discrepancies are unclear, but are likely to be associated with variations in cell systems and experimental conditions used. Thus, while a possible general model seems to point toward H2Aub being largely independent of H3K27me3, definitive answers on the role of H3K27 methylation in shaping H2A ubiquitination are still needed.

*Role of H2Aub in H3K27 methylation*: Inactivation of PRC1 in Drosophila, which results in the global depletion of H2Aub, also leads to partially decreased levels of H3K27me3 in mutant embryos^19,26^. Interestingly, H2Aub creates a docking site for PRC2 complex containing Jumonji And AT-Rich Interaction Domain Containing 2 (JARID2), and this leads to H3K27 trimethylation^83^ (section “Readers of H2Aub”). This provides a molecular link between H2Aub and H3K27me3 that might also explain the co-existence of both marks on PcG target genes.

In mammalian systems, notably mESC, inhibition of H2Aub deposition leads to a substantial reduction of PRC2 recruitment and concomitant deposition of H3K27me3 at PcG targets^13–16,32,84^. In addition, inhibition of PRC1 and subsequent H2Aub deposition prevents the recruitment of the PRC2 component EED (Embryonic Ectoderm Development) and subsequent H3K27 trimethylation during X chromosome inactivation^85^. This feedback loop between these two histone marks seems also to be mediated by JARID2 in mammals^83,86^. Consistent with this model, depletion of H2Aub results in a strong reduction in the chromatin occupancy of JARID2^15,16,87^. However, inactivation of JARID2 in mESC results in a marginal reduction of the global levels of H3K27me3, although this can be explained by functional redundancy within PRC2 complexes^87^. Strikingly, other studies showed that ablation of PRC1 does not impact H3K27me3^30,87,88^, which is difficult to reconcile with the findings mentioned above. While discrepancies can be explained by differences in experimental settings or tissue-specific regulation, additional studies are needed to further define how deposition of H2Aub impacts H3K27me3 distribution.

In summary, PRC1 recruitment to gene regulatory regions and subsequent H2Aub deposition is highly influenced by DNA-binding proteins, PRC1 subunit composition and the state of chromatin. While several scenarios of crosstalk between H2Aub and H3K27me3 can be envisioned, one emerging model involves the deposition of H2Aub by vPRC1 leading to the recruitment of PRC2 and subsequent deposition of H3K27me3. Methylation of H3K27, in turn, leads to the recruitment of cPRC1 through methyl binding by the CBX subunits. This model also implies different functions between vPRC1 and cPRC1 complexes (discussed later). These findings also raise some interesting questions of what determines the composition and stoichiometry of PcG complexes and how different complexes dynamically direct target gene expression outcomes. The use of quantitative approaches to analyze H2Aub, H3K27me3 and other histone PTMs throughout the genome in well-defined cell models and experimental settings would help unify conclusions.

### Structural insights into the mechanism of H2A ubiquitination by PRC1

While the PRC1 complex was originally purified from Drosophila cells and shown to antagonize the chromatin-remodeling activity of a SWI/SNF factor^89^, it was later purified from human cells by virtue of its ability to ubiquitinate H2Aub^9^. Subsequently, a mammalian PRC1 complex was reconstituted in vitro, which revealed that BMI1 (PCGF4) strongly enhances the E3 ligase activity of RING1B and is essential for complex assembly^17^. In addition, early structural studies indicated that RING1B and BMI1 form a heterodimer via their RING domains while the E2 ubiquitin conjugating enzyme UBCH5 interacts directly with RING1B^90,91^. The crystal structure of PRC1 ubiquitination module (composed of the E2 UBCH5C and the minimal RING1B/BMI1 RING heterodimer) indicates that the N-terminal tail of RING1B is wrapped around BMI1 stabilizing the RING-RING interaction and simultaneously promoting E2 binding^90,91^. **(**Fig. 2c**)**. On the other hand, the positioning of the RING1B/BMI1 heterodimer on the nucleosome core particle (NCP) involves an interface composed of basic residues that interact with the nucleosome acidic patch, an electronegative cleft formed by H2A/H2B histones dimer that constitutes a protein interaction interface^92,93^. Interestingly, all four histones contribute to the interaction with the RING1B/BMI1 complex. Of note, while the RING1B/BMI1-NCP interaction is independent of the DNA sequence, the proper orientation of RING1B/BMI1 heterodimer towards H2A involves an additional interaction of the E2 enzyme with nucleosomal DNA^93^. This multiple surface binding of the RING1B/BMI1/UBCH5C results in an enhanced catalytic activity of the complex while positioning the E2 enzyme very closely to H2A^93^. The fact that RING1B/BMI1-UBCH5C interacts with H2A-H2B, H3-H4, and DNA explains why PRC1 requires a fully assembled nucleosome for ubiquitination^93^. Overall, these studies provided important insights into the mechanism of H2A ubiquitination that involve the additive effects of multiple interactions that altogether increase the overall affinity and specificity of PRC1 towards nucleosomal H2Aub^92,93^.

## Deubiquitination of H2Aub by PR-DUB and BAP1

**Fig. 3:**
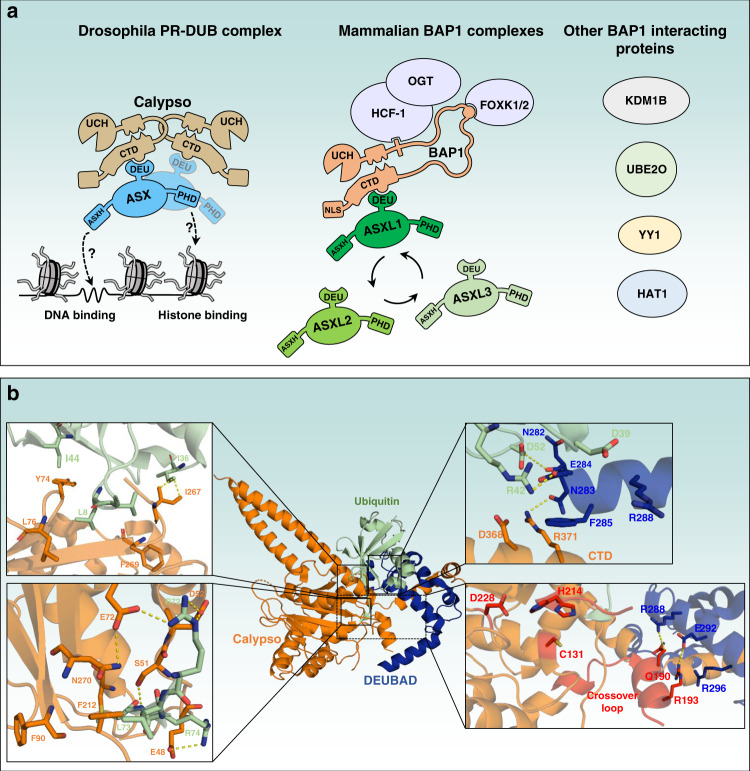
PR-DUB and BAP1 as major deubiquitinase complexes for H2Aub. **a** Schematic representation of the Drosophila PR-DUB (Calypso/ASX) and mammalian BAP1 complexes (BAP1/ASXLs). The dotted arrows indicate potential interactions of ASX with chromatin. Calypso and ASX form an oligomer of 2:2 molecules. This specific arrangement is important for the recruitment to chromatin and H2Aub deubiquitination. Note that the mammalian PR-DUB complex (BAP1 complexes) is composed of many additional subunits including HCF-1, OGT, and FOXK1/2. Other BAP1-interacting partners, KDM1B, UBE2O, YY1 and HAT1 are shown. The arrows in circle indicate the mutually exclusive binding of ASXLs to BAP1. **b** Structural model of the Drosophila core PR-DUB complex (Calypso/DEUBAD (DEUBAD of Asx)) bound to ubiquitin. The model was obtained by superimposing the crystal structure of Calypso/DEUBAD (PDB: 6HGC) with the UCH-L5-RPN13-Ub crystal structure (PDB: 4UEL). Hydrogen bound interactions between calypso catalytic domain and ubiquitin are shown (left panels). DEUBAD also makes contact with the CTD domain of Calypso and ubiquitin (right top panel). The DEUBAD interaction with Calypso stabilizes Calypso’s crossover loop (shown in red, right bottom panel) which promotes Calypso’s catalytic activity towards ubiquitin. The triad catalytic sites: C131, H214, D228 are shown in red (right bottom panel).

The H2Aub DUB Calypso was originally identified as a gene whose mutation induces a PcG phenotype in Drosophila^94^. BAP1, the mammalian orthologue of Calypso, is found in the cytoplasm and nucleus^95,96^. Nuclear BAP1 associates with ASXLs and also catalyzes H2Aub deubiquitination^38,97^ **(**Fig. 3a**)**. Of note, depletion of ASXL1 and ASXL2 strongly reduces BAP1 protein levels indicating the importance of these factors in regulating BAP1 function^97^. In addition, nuclear BAP1 assembles, with different stoichiometries, large complexes containing several transcriptional regulators including, the Host Cell Factor-1 (HCF-1), the O-Linked N-Acetylglucosamine Transferase (OGT), the Lysine demethylase KDM1B (LSD2/AOF1), the Histone acetyltransferase HAT1 as well as FOXK1/2, YY1 transcription factors and the E2/E3 hybrid enzyme UBE2O^98–100^ (Fig. 3a**)**. While BAP1, HCF-1, OGT, ASXLs, and possibly FOXK1/2, appear to form the core complex^95,97,100–102^, the composition of BAP1 subcomplexes remains to be rigorously determined. In addition, how BAP1 complexes catalyze H2Aub deubiquitination in different physiological contexts and during development remains to be established.

Mechanistically, Calypso/BAP1 DUB activity requires a stable interaction with a conserved domain, termed DEUBAD (DEUBiquitinase ADaptor) in Asx and ASXLs^38,97,103,104^. BAP1 adopts a conformational configuration through its association with the DEUBAD forming a Composite Ubiquitin-Binding Interface (CUBI). This interface is essential for BAP1 binding and deubiquitination of H2Aub^97^.

Structural studies of ubiquitin bound to a minimal PR-DUB complex indicated that BAP1/Calypso catalytic domain engages with ubiquitin through the hydrophobic patch and the C-terminal tail of ubiquitin^103,105^ **(**Fig. 3b**)**. The overall model for PR-DUB-ubiquitin also revealed a third binding mode between BAP1/Calypso and ubiquitin, which involves the DEUBAD. The DEUBAD increases Calypso-ubiquitin binding by favouring a suitable arrangement between the C-terminal domain (CTD) and the UCH (Ubiquitin C-terminal Hydrolase) catalytic domain^103,105^. This includes a stabilization of the crossover loop (an amino acid extension that limits substrate access to the active site) and direct interactions between the DEUBAD and ubiquitin^103,105^ **(**Fig. 3b**)**. Another layer of stabilization consists of the interaction between the DEUBAD and the crossover loop of BAP1/Calypso. Overall, the proposed model suggests that DEUBAD enhances BAP1/Calypso activation by increasing the latter’s affinity for ubiquitin, through conformational changes. Remarkably, Calypso-DEUBAD are arranged in a specific oligomerization state formed by 2:2 Calypso:DEUBAD molecules^103,106^. This oligomerization requires the coiled-coil domain of both Calypso molecules and involves the formation of an extended cleft separating the UCH domains. This arrangement facilitates the recruitment on nucleosomes, as dimerization defective mutants of Calypso exhibit reduced PR-DUB interaction with nucleosomes^106^. Importantly, oligomerization of Calypso is only required for its activity towards nucleosomal histone H2A, as the intrinsic DUB activity is not altered by mutation of the dimerization interface^106^.

Interestingly, the DEUBAD domain itself is predominately monoubiquitinated and this event is conserved from Drosophila to human^104,107^. DEUBAD monoubiquitination enhances H2Aub deubiquitination by BAP1/Calypso, providing another layer of regulation of H2Aub deubiquitination^104,107^. Conversely, it was recently reported that methylation of adenine N6 in DNA (N^6^-methyladenine) is recognized by the ASXH domain of ASXL1, triggering the degradation of BAP1/ASXL1 and preventing H2Aub deubiquitination and gene silencing^108^. Hence, it will be interesting to decipher how deubiquitination of H2Aub is orchestrated in response to stimuli and signaling pathways.

## Readers of H2Aub

**Fig. 4:**
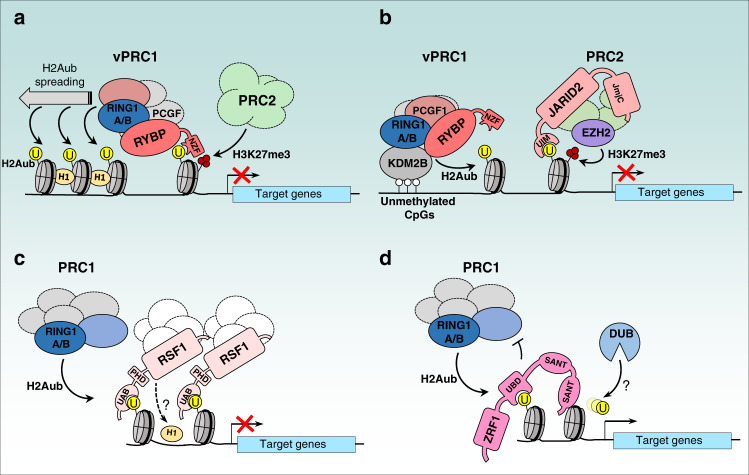
H2Aub readers in transcription regulation. **a** The vPRC1 component RYBP uses its NZF-UBD to bind H2Aub. This binding is increased with nucleosome compaction with histone H1 to promote H2Aub spreading and a repressive state of chromatin. **b** Positive-feedback loop between PRC1 and PRC2 complexes is ensured by the binding of vPRC1 component, KDM2B to unmethylated CpGs island promoting H2Aub deposition. This results in a specific binding of nucleosomal H2Aub by the PRC2 component JARID2 through its UIM domain. JARID2 binding promotes the recruitment of the PRC2 complex and eventually the establishment of H3K27me3 by EZH2 and maintenance of gene repression. **c** RSF1 binds H2Aub through its UAB domain and promotes the recruitment of the RSF1 chromatin-remodeling complex. This ensures proper nucleosome arrangement at target gene promoters with maintenance of histone H1 positioning, ensuring gene transcription repression in collaboration with PRC1 complexes. **d** ZRF1 uses its UBD Ubiquitin-Binding Domain to bind H2Aub, thus blocking the access of PRC1 to the nucleosomes, and likely promoting recruitment of DUBs, allowing ubiquitin removal and transcription activation. Ubiquitin is shown as a yellow circle and H3K27me3 is shown by the three red circles.

Several factors including RYBP, JARID2, the Zuotin-Related Factor (ZRF1) and the Remodeling and Spacing Factor 1 (RSF1) have been proposed as readers for H2Aub^85,86,109–112^ (Fig. 4). RYBP plays an important role in the deposition of H2Aub and genome-wide binding of RYBP is strongly reduced following depletion of RING1A/RING1B in mESCs^16,42,45,112^. In defining how RYBP interacts with chromatin, it was initially found that RYBP can bind ubiquitin and H2Aub through a NZF (Nuclear protein localization 4 Zinc Finger) ubiquitin-binding domain^84,109^ (Fig. 4a). On the other hand, RYBP was also shown to act as an activator of PRC1^42,84,109^. However, in the latter studies, the impact of RYBP-H2Aub binding was not clearly separated from its E3 ligase stimulatory effect^42,84^. Interestingly, vPRC1 complexes containing PCGF3/5 promote Xist RNA-dependent X chromosome inactivation through RYBP-H2Aub binding^85^. Notably, following Xist induction in RYBP knockout mESCs, RYBP, but not its H2Aub-binding defective mutant (T31A/F32A), rescues PcG-mediated deposition of H2Aub and H3K27me3 on inactive X chromosome^85^. More recently, it was revealed that RYBP-mediated H2A ubiquitination is strongly increased on partially ubiquitinated versus unmodified nucleosome arrays^112^. This stimulatory effect is dependent on the ability of RYBP to bind H2Aub and is increased by nucleosome array compaction with histone H1. Moreover, using a gene reporter assay, it was observed that H2Aub is propagated in vivo through the ability of RYBP to bind H2Aub, thus providing an epigenetic mechanism for H2Aub maintenance during cell division^112^. In summary, RYBP appears to ensure a positive-feedback mechanism by promoting H2Aub propagation and spreading. Moreover, the role of this factor in allosteric regulation of PRC1 might also be orchestrated with its H2Aub reader function. Structural studies of PRC1-RYBP bound to nucleosomes should provide insights into H2Aub deposition and propagation by RYBP. On the other hand, taking into account that H2Aub is erased during mitosis and deposited in G1^113^, and that the half-life of H2Aub is relatively short^114,115^, the mechanism by which RYBP would contribute to the global maintenance of H2Aub is perhaps more complex than anticipated and further studies are required to better define the dynamics of H2Aub deposition and removal.

JARID2 promotes PRC2-EZH2 enzymatic activity through its N-terminal domain^116^. Insights into the mechanism of activation were provided by structural studies showing that JARID2 stabilizes the PRC2 complex and allosterically promote PRC2-mediated H3K27 methylation^117–120^. It was also found that JARID2 uses its N-terminal ubiquitin interaction motif (UIM) to specifically bind nucleosomal H2Aub and this results in increased H3K27 trimethylation, thus defining a positive-feedback loop between PRC1 and PRC2 **(**Fig. 4b**)**^83,86^. These findings are in agreement with several reports, demonstrating the dependency of PRC2 recruitment on H2A ubiquitination^13,15,63^, although further studies on the link between H2Aub binding by JARID2 and H3K27me3 deposition by PRC2 are needed. Notably genome-wide studies of both histone PTMs, following gene replacement in vivo, using discrete mutations of JARID2 in its UIM will help elucidating the link between H2Aub and H3K27me3. It will also be interesting to further dissect, at the structural level, how JARID2 binding to H2Aub is linked to its stimulatory effect of EZH2 methyltransferase activity.

The remodeling and spacing factor RSF1 was also recently proposed as a reader of H2Aub^111^. ChIP-seq analysis showed an overlap between RSF1 and H2Aub peaks and depletion of RING1B appears to reduce RSF1 chromatin recruitment. Knockdown of RSF1 induces target gene derepression without alteration of RING1B protein levels or H2A ubiquitination state^111^. A model was proposed that implicates the binding of RSF1 to H2Aub in order to promote nucleosomal stability and facilitate transcriptional silencing^111^ (Fig. 4c). However, it remains unclear how RSF1 stabilizes chromatin through H2Aub binding and how RSF1 interacts with PcG factors to ensure repression.

In contrast to the transcription silencing effects of the factors described above, ZRF1 was found as a potential reader of H2Aub with the opposite outcome^110^. ZRF1 binds directly to H2Aub, through its conserved zuotin domain, and appear to compete with PRC1 on many of its target genes^110^. Following retinoic acid induction, over half of ZRF1 genomic targets overlap with those of RING1B and H2Aub. The transcriptional activation of the overlapping targets was shown to be dependent on the presence of ZRF1, suggesting that the recruitment of this factor facilitates derepression of PRC1 targets **(**Fig. 4d**)**^110^. It will be interesting to further dissect the mechanism that orchestrates ZRF1 recruitment and its link with H2Aub deubiquitination and gene derepression. ZRF1 would likely rely on additional interacting factors to efficiently bind H2Aub and compete with PRC1. The potential identification of these factors as well as structural studies on ZRF1-chromatin interaction might provide insights into the mechanisms of gene activation by ZRF1.

In summary, several factors were proposed as readers of ubiquitinated H2A. However, a deeper understanding of their contributions in PcG silencing or gene activation will require further studies. In particular, additional questions relate to the chromatin contexts and the signaling events that might dictate which potential H2Aub reader needs to be recruited to coordinate gene expression.

## Functions of H2Aub in chromatin-associated processes

### Roles of H2Aub in transcriptional repression

**Fig. 5:**
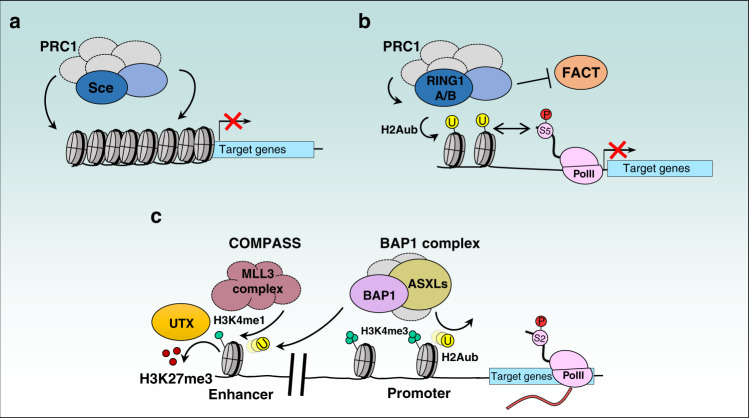
Regulation of gene transcription by H2Aub ubiquitination and deubiquitination. **a** Recruitment of PRC1 complexes to compact chromatin regions in H2Aub-independent manner. These chromatin domains become inaccessible to the transcriptional machinery and are thus repressed. **b** H2A ubiquitination could contribute to gene repression by blocking the FACT histone chaperone. This would block nucleosome rearrangement resulting in maintenance of poised RNA PolII at promoters and inhibition of transcription elongation of specific target genes. Poised PolII is phosphorylated on serine 5. **c** BAP1 DUB complexes act at enhancers to recruit MLL3 and UTX to deposit H3K4me1 and demethylate H3K27me3, respectively. These events along with deubiquitination of H2Aub promote gene activation. Ubiquitination is shown in yellow circle, H3K27me3 is shown by the three red circles, H3K4me1/3 is shown by green circles and phosphorylation by bigger red circle.

Inactivation of PRC1 E3 ligase activity in Drosophila, through the use of a Sce-E2 ubiquitin conjugating-interaction defective mutant (I48A), results in the depletion of H2Aub, but has no visible effects on the expression of the Hox genes Antennapedia (Antp), Ultrabithorax (Ubx), and Abdominal-B (Abd-B)^19^. However, the complete absence of Sce induces major embryotic homeotic transformations associated with deregulation of Hox gene expression^19^. In addition, replacing histone H2A gene cluster with an ubiquitination-defective mutant allele leads to similar conclusions that absence of H2Aub does not impact Hox gene expression^19^. These results are consistent with previous studies showing that PRC1 promotes chromatin compaction independently of its catalytic activity^89,121^. Interestingly, loss of PRC1 during Drosophila embryonic development results in higher order opening of chromatin at Hox loci^122^. Overall, while H2Aub is still required for viability of Drosophila embryos, the presence of Sce, but not its catalytic activity, is required to maintain Hox gene repression (Fig. 5a), indicating that PRC1 possess H2Aub-dependent and independent chromatin-associated activities.

In mammalian cells, initial studies using E2-interaction defective mutant of RING1B (e.g., I53A), provided initial evidence that enzymatic activity of RING1B is important for PcG gene silencing and maintenance of mESC identity^28^. However, RING1B I53A mutation does not completely abolish the E3 ligase activity^15,16,123^. Using RING1B mutants completely defective in E3 ligase activity (e.g., I53A/D56K), it was shown that PRC1 catalytic activity is required for neuronal gene silencing in neural stem or progenitor cells^123^. Using similar approaches in mESCs, it was later shown that PRC1-mediated ubiquitination is critical for repression of PcG target genes^15,16^. Thus, PRC1 catalytic activity plays an important role in transcriptional repression in higher organisms. Mechanistically, H2Aub may interfere with the function of RNA Polymerase II (RNA Pol II). In mESC, depletion of both RING1A/RING1B induces a loss of H2Aub and release of poised RNA Pol II, and this was associated with transcription elongation and increased gene expression^124^ (Fig. 5b). On the other hand, H2A ubiquitination appears to inhibit the recruitment of the histone chaperone FACT (Facilitates Chromatin Transcription), thus blocking nucleosomal rearrangement and subsequent early gene transcription elongation^125^ (Fig. 5b). However, it remains largely unclear how H2Aub inhibits FACT recruitment and whether this involves intermediate factors harboring ubiquitin-binding domains.

On the other hand, depletion of the PR-DUB complex in Drosophila results in the global increase of H2Aub levels and this was unexpectedly associated with transcriptional derepression of Ubx, in a DUB activity-dependent manner^38^. While the exact mechanism by which Calypso represses transcription remains elusive, these results support the notion that ubiquitination and deubiquitination of H2A might be dynamically involved in regulating Hox gene expression^38^. Moreover, this raises the question of whether mammalian BAP1 can also possess transcription repression properties. In investigating its potential functions in gene expression, BAP1 was initially described as a transcription activator^100^. BAP1 co-activator function correlated with increased H3K4me3 and concomitant depletion of H2Aub and H3K27me3 at target genes^126–129^. Consistent with this, BAP1 was also shown to promote the recruitment of KMT2C (MLL3) and KDM6A (UTX) to enhancers, thus ensuring H3K4me1 deposition and H3K27me3 removal, respectively^130^ (Fig. 5c). Of note, BAP1, but not Calypso, possesses a larger insertion in the middle of the protein that is responsible for interaction with HCF-1, OGT, and FOXKs^38,97,131^ (Fig. 3a). Notably HCF-1 is part of TrxG complexes^2^, and this might explain BAP1 transcription activation properties that would be coordinated with H2A deubiquitination. Moreover, FOXKs were shown to play an important role in BAP1-mediated H2Aub deubiquitination and transcriptional regulation^129,131,132^. Overall, while the general consensus is that BAP1-mediated H2A deubiquitination in mammalian systems promotes transcriptional activation^127–129^, there is also evidence for BAP1-mediated H2Aub deubiquitination promoting transcriptional repression^126,132,133^. Further studies are clearly needed to determine whether mammalian BAP1 has dual transcriptional regulatory functions and what are the exact cellular and chromatin contexts that involve one function versus another.

### Does H2Aub play a role in transcriptional activation?

Intriguingly, gene repression was not the only outcome associated with the presence of H2Aub, as recent studies implicated PRC1 in gene activation^134–136^. For instance, during mESC differentiation, a transient swap of CBX7 with CBX8 subunits of PRC1 complex was shown to promote transcription activation of genes that appear to maintain a certain level of H2Aub^135^. However, mESCs undergoing differentiation are heterogeneous cell populations and RING1B or H2Aub ChIP signals detected at target genes might be contributed by non-differentiated cells. vPRC1 (PRC1.5) containing the casein kinase 2 (CK2), AUTS2 and PCGF5 subunits was also recently implicated in gene activation^134^. AUTS2 appears to block H2A ubiquitination by recruiting CK2 which was shown to phosphorylate and inactivate RING1B. In parallel, AUTS2 could also interact with and recruit the acetyltransferase p300 to promote gene expression^134^. However, these studies relied on transcription reporter systems and further validation is needed to evaluate the function of PRC1.5 in the context of natural recruitment to target genes^134^. In addition, it is unclear how the proposed function of PRC1.5 can be reconciled with the recently described PCGF5 function in H2Aub deposition and X chromosome inactivation^32,85^. Similarly, transcription activation in quiescent B and T lymphocytes also appears to be a manifestation of PRC1’s counterintuitive activity. Both the Aurora B kinase and the PRC1 complex appear to be recruited to actively transcribed genes^136^. In this case, Aurora B was shown to phosphorylate the E2 ubiquitin-conjugating enzyme UBCH5C to prevent RING1B-mediated H2A ubiquitination. However, the exact molecular mechanism of gene activation remains unclear^136^. In addition, the H2Aub-independent function of PRC1 was mostly associated with gene repression and chromatin compaction^121,137,138^. Thus, it remains to be determined, at the mechanistic level, how PRC1 complex composition can influence gene activation.

Clearly, additional work is needed to better define how H2Aub exactly regulates transcription and which chromatin contexts and factors act in concert or antagonism with this modification in the regulation of gene expression.

### Role of H2Aub in DNA damage signaling and repair

The cellular response to genotoxic stress involves multiple signaling and repair factors, many of which are associated with chromatin function. Notably, the early responders, ATM (Ataxia Telangiectasia-Mutated) and ATR (ATM- and Rad3-related) kinases, phosphorylate several factors, including the histone variant H2AX, leading to the ordered assembly of multi-protein complexes that ensure DNA damage recognition and execution of the repair process^139–142^. It was initially found that UV-induced helix-distorting DNA lesions promote the local recruitment of RING1B and subsequent ubiquitination of H2A in ATR-dependent manner^143^. The initial incision at the DNA damage site by the nucleotide excision repair (NER) machinery appears to be required for H2Aub deposition at DNA damage sites, although the precise impact of H2Aub on the execution of the NER process remains unknown^143^.

**Fig. 6:**
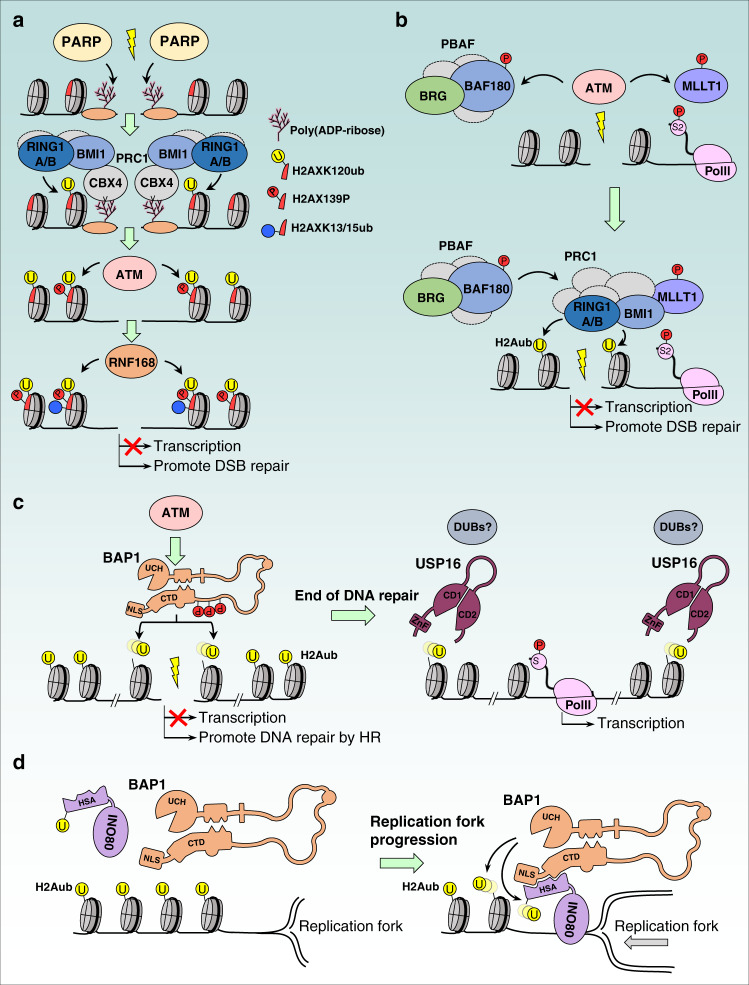
Involvement of H2Aub in DNA double strand break signaling and replication fork progression. **a** Induction of DNA double strand breaks (DSBs) induces a rapid recruitment of PARP to DNA damage sites and subsequent protein poly(ADP-ribosyl)ation. Poly(ADP-ribose) serves as an anchor for the PRC1 complex, through its CBX4 subunit which leads to the recruitment of PRC1 to DNA damage site and ubiquitination of the histone variant H2AX. This ubiquitination event induces a more efficient recruitment of ATM and phosphorylation of H2AX (γH2AX), a hallmark of the DNA damage response. This in turn leads to the recruitment of RNF168 with catalyzes H2AXK13/K15 ubiquitination. Altogether, this cascade of posttranslational modifications leads to effective recruitment of the DNA repair machinery and inhibition of transcription. **b** Specific recruitment of the PRC1 complex to DNA DSB sites could be enhanced through interaction between BMI1 and the phosphorylated form of the transcription elongation factor MLLT1. Phosphorylation of PBAF also promotes the recruitment of the PRC1 complex to the site of DNA damage. **c** BAP1 is phosphorylated following DNA damage and is also recruited to DSB site to promote homologous recombination (HR). BAP1 appears to deubiquitinate H2Aub only near the DSB site, likely promoting DNA-end resection. Once the repair process is completed, the deubiquitinase USP16 or other DUBs could remove H2Aub mark more widely, allowing normal gene transcription. CD1 and CD2: the two parts of the catalytic domain. **d** During DNA synthesis, the chromatin-remodeling factor INO80 is protected from proteasomal degradation by BAP1. On the other hand, BAP1 deubiquitinates H2A and promotes coordinated DNA replication. HSA, Helicase-SANT associated domain. Ubiquitination is shown by the yellow circle, and phosphorylation by the red circle.

The role of H2Aub in DNA damage signaling has been mostly examined in the context of DNA double strand breaks (DSBs). Following induction of DSBs and phosphorylation of H2AX (γH2AX), an orchestrated ubiquitin-signaling cascade involving the ubiquitin ligases RNF8 and RNF168 leads to the monoubiquitination of H2AK13/K15 followed by the recruitment of 53BP1 and BRCA1 repair factors and DSB repair^141,144^. Using laser micro-irradiation-induced localized DNA damage, it was found that BMI1 and RING1B are rapidly and transiently recruited to DSB sites to promote the ubiquitination of γH2AXK120ub (γH2AXK120 is the corresponding ubiquitination site of H2AK119) and subsequent assembly of DNA repair factors^145^. These events are dependent on PARP-mediated poly(ADP-ribosyl)ation, which occurs rapidly following DNA break formation^145–147^. The initial recruitment of BMI1 and RING1B occurs through the binding of CBX4 to poly(ADP-ribose) prior to noticeable phosphorylation of H2AX and RNF8/RNF168 signaling^145,147^ (Fig. 6a). Thus, DNA damage induces a rapid recruitment of PcG factors and γH2AXK120 ubiquitination, which facilitates H2AK13/K15 ubiquitination and DSB signaling^145,147^. Of note, in many studies on DNA damage signaling, H2AK119ub (H2Aub) and H2AXK120ub (H2AXub) cannot be distinguished by immunostaining or ChIP, as both PTMs are recognized by antibodies against these PTMs^148–151^. Therefore, the specific contribution of each of these histone marks in DNA damage signaling and repair remains unclear.

Mechanistically, it is still not well-defined how γH2AXub or H2Aub promotes DNA repair, but an attractive hypothesis that was originally proposed consists of the involvement of H2Aub/γH2AXub in transcription repression of regions neighboring DNA damage in order to facilitate the repair process^145,146^. Indeed, genomic regions around DSB sites become silenced in an ATM-dependent manner (Fig. 6a)^148^. When BMI1/RING1B is inhibited, ATM-dependent transcriptional silencing no longer occurs^115^. Interestingly, the chromatin-remodeling complex PBAF, which also acts downstream of ATM, appears to mediate transcriptional silencing in response to DSBs through PRC1/PRC2 recruitment^149^. The presence of the PBAF complex and ATM-mediated phosphorylation of its subunit BAF180 appears to be required for efficient deposition of H2Aub/γH2AXub and rapid DNA repair (Fig. 6b). On the other hand, phosphorylation of the transcriptional elongation factor MLLT1, component of the Super Elongation Complex (SEC), by ATM enhances its interaction with BMI1^150^. MLLT1 would normally promote transcription elongation. Instead, its phosphorylation promotes the subsequent recruitment of the PRC1 complex to DNA damage sites, H2Aub/γH2AXub deposition and transcriptional silencing (Fig. 6b)^150^. How PBAF and MLLT1 functions are coordinated at the DSB sites remains an interesting question to address.

The roles of H2Aub/γH2AXub in the DNA damage response were also revealed through investigating the contribution of DUBs in DSB repair. BAP1 promotes DSB repair, likely as a part of its tumor suppressor function^96,151,152^. BAP1 is phosphorylated by ATM, which promotes its recruitment to the DSB site. BAP1 might subsequently remove ubiquitin from H2Aub/γH2AXub, but only at the vicinity of the DSB site^151^. BAP1 might facilitate chromatin organization near DSB sites, and this might lead to the initiation of DNA-end resection and subsequent DSB repair by homologous recombination^151,152^ (Fig. 6c). USP16 would remove H2Aub/γH2AXub following the accomplishment of DNA repair promoting the recovery of gene transcription^148^ (Fig. 6c), although a direct role of USP16 in DSB signaling has been recently debated^153^. Despite these findings, the exact mechanism behind DUB recruitment and removal of ubiquitin from H2Aub/γH2AXub at DSB sites is not known. In addition, the temporal order of DUB recruitment at DSB sites and their specific roles in DNA damage signaling and repair require further studies.

In summary, while the importance of H2Aub/γH2AXub in DNA damage signaling and repair processes is becoming increasingly established, a mechanistic understanding is needed to explain how this histone PTM acts in concert with other histone modifications and chromatin-associated complexes to orchestrate transcription and DNA repair at DNA damage sites.

### Roles of H2Aub in other DNA-associated processes

H2Aub was also implicated in other DNA-associated processes including DNA replication and chromosome segregation. Early observations revealed that H2Aub could form distinct foci which partially colocalized with Proliferating Cell Nuclear Antigen (PCNA)^154^. This colocalization was enhanced following inhibition of DNA replication, suggesting a potential regulatory mechanism involving H2Aub at replication forks^154^. It was also shown that the major satellite regions at pericentric heterochromatin—a condensed and silenced form of chromatin found near centromeres and is characterized by hypoacetylated histones and the presence of H3K9me3 ― are decorated with H2Aub^12^. It was proposed that H2Aub might be involved in the progression of the replication fork in these heterochromatic regions^12^. Recently, it was found that INO80 chromatin-remodeling factor associates with BAP1, which appears to promote INO80 recruitment to replication forks^155^. In addition, BAP1 also targets INO80 for deubiquitination thus promoting its stability at the replication fork (Fig. 6d**)**^155,156^. It was proposed that the presence of H2Aub provides an anchor-like function to recruit BAP1/INO80 to ensure proper replication fork progression and genomic stability^155,156^. However, how the dynamics of H2Aub deposition and removal impacts the progression of the replication fork remains largely unknown.

Changes in H2Aub levels were also associated with mitotic progression. It was previously observed that H2Aub levels are strongly reduced at the onset of M phase and re-established in early G1^113,157^. Depletion of USP16 was shown to prevent the mitotic decrease of H2Aub levels and inhibit cell cycle progression^158^. Moreover, H2Aub levels were reported to be inversely correlated with histone H3S10 phosphorylation by Aurora B kinase hinting to a potential crosstalk between these two histone modifications^158^. Mechanistically, USP16 appears to directly deubiquitinate H2Aub and this was shown to influence histone H3S10 phosphorylation (H3S10P), chromosome segregation and mitotic progression^158^. However, it was recently shown that H2Aub deubiquitination does not precede H3S10P deposition^153^. Thus, the link between phosphorylation of H3S10 and deubiquitination of H2Aub remains unclear.

## Deregulation of H2Aub in human pathologies

Several components of the PRC1 complexes, including RING1B and BMI1, are overexpressed in cancer^159–163^. Upregulation of BMI1 is associated with increased proliferation, reduced apoptosis and increased capacity of stem cell self-renewal^163–166^. The oncogenic properties of BMI1 appear to involve its transcriptional repressor function. BMI1 is a negative regulator of several target genes controlling cell cycle progression such as p16^Ink4a^, p19^Arf^, p21^WAF1/CIP1^, in addition to the tumor suppressor PTEN^160,163,167–169^. However, oncogenic functions of BMI1 that are independent of H2Aub have also been proposed^162,170^. On the other hand, there are also evidence for tumor suppressor activities for some PRC1 components including PHC3 and KDM2B^171,172^. Importantly, the relationship between the function of several PRC1 proteins and H2Aub in tumorigenesis remains unclear.

The potential role of H2Aub and cancer is also provided by the DUB BAP1 and its co-factors ASXLs. BAP1 is a tumor suppressor mutated in multiple cancers^101,173–182^. Cancer mutations of the catalytic domain compromise the DUB activity of BAP1^181^. Interestingly, a cancer mutation consisting of a small in-frame deletion in the CTD domain of BAP1 results in its selective dissociation from ASXLs and inability to deubiquitinate H2Aub and regulate cell proliferation^97^. In further linking BAP1-mediated H2A deubiquitination and tumor suppression, it was found that loss of BAP1 results in the repression or activation of genes that regulate cell death^126,128^. While the role of H2Aub in repression versus activation has not been directly tested in these studies, the data suggest that catalytic activity of BAP1 regulates cell death and tumor suppression^126,128^. BAP1 inactivation also compromises its ability to promote DNA repair and genomic integrity^96,151,152,156^, although it is currently unclear what is the contribution of BAP1 function in DNA repair for tumor suppression.

**Fig. 7:**
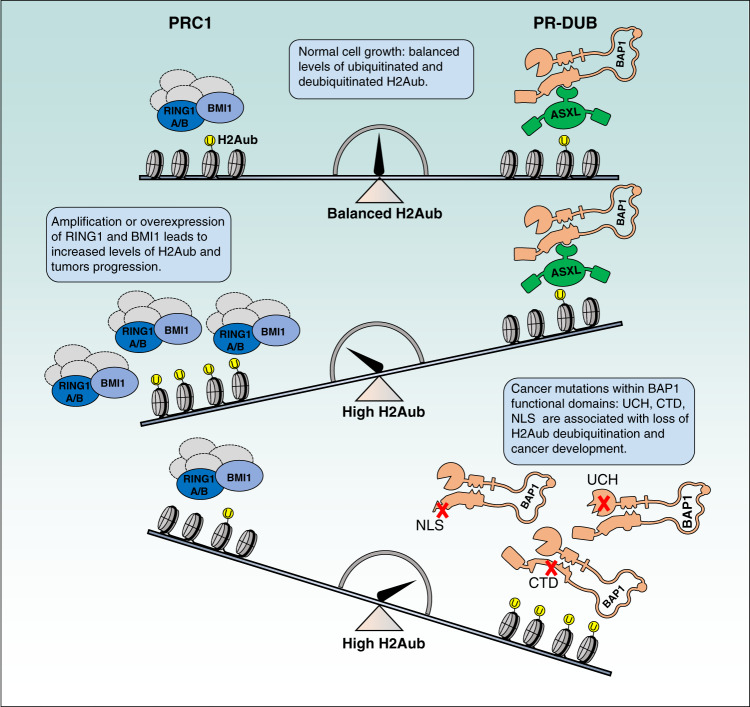
Model for the potential role of H2Aub in cancer development. PRC1 mediates H2Aub deposition and represses target genes that are negative regulators of cell cycle progression and cell death. PRC1 also promotes stem cell self-renewal. PRC1 overexpression results in increased cell ability to overcome cell cycle control, reduced apoptosis as well as increased capacity of stem cell self-renewal. BAP1 regulates cell proliferation and promotes cell death. Inactivation of BAP1 results in increased H2Aub deposition at target genes and modulation of cell cycle and resistance to cell death. PRC1 and BAP1 also regulate DNA repair, defect of which can promote carcinogenesis. BAP1 cancer-associated mutations disrupting BAP1 functional domains are shown by the red cross. Ubiquitination is shown by the yellow circle.

Overall, an emerging model of H2Aub-associated tumorigenesis involves the gain of function of PRC1 or the loss of function of PR-DUB with the resulting increase of H2Aub levels on chromatin. Uncontrolled H2Aub deposition on chromatin might be a key determinant in cancer development (Fig. 7). However, despite the number of studies indicating an important role of BAP1 in tumor suppression, recent studies also provided evidence for a potential gain of function of BAP1/ASXLs in myeloid malignancies^29,107,183^. Thus, further studies are needed to firmly determine how altered deposition of H2Aub exactly alters chromatin function and promotes oncogenic signaling.

Mutations in several components implicated in modulation of H2Aub levels have been associated with several syndromes and brain pathologies. For instance, mutations in AUTS2 are associated with autism and intellectual disability^184^. Similarly, defects in PHC1 are linked to brain development disorders such as autosomal recessive primary microcephaly. PHC1 mutations are associated with impaired H2A ubiquitination, cell cycle progression and DNA repair^185^. PR-DUB complex is also associated with rare developmental syndromes. For example, germline mutations of ASXL1 are found in patients with congenital disorders such as the Bohring-Opitz Syndrome, a very rare syndrome characterized by multiple congenital malformations and severe intellectual disabilities^186^. ASXL2 and ASXL3 mutations are also linked to developmental disorders^187,188^. Notably, Bainbridge-Ropers syndrome patients with mutated ASXL3 exhibit higher levels of H2Aub which correlated with differential gene expression outcomes^189^. Nonetheless, the exact mechanisms that mediate the effects of ASXLs mutations in the pathogenesis of these diseases are largely unknown. Overall, defects in either components that establish or erase H2Aub are observed in disease conditions, suggesting an important role of this PTM in normal physiology. The possibility to conduct genome-wide mapping for this modification, in conjunction with gene expression analysis and functional assays following gene targeting, would help to elucidate how deregulation of H2Aub underlies human disease.

## Concluding remarks

Almost half a century of research on H2Aub revealed important aspects of its function. However, how H2Aub coordinates cellular processes and how its deregulation contributes to human disease remains incompletely defined. At the mechanistic level, how H2Aub deposition is translated into changes of chromatin function that can further impact transcription or DNA repair remains to be fully established. A major knowledge gap also remains regarding the regulation of H2Aub. In particular, PRC1 complexes are targeted by PTMs and it is unclear how these PTM translate signaling pathways into changes in H2Aub levels and chromatin function. On the other hand, recent studies provided possible mechanisms of H2Aub propagation. However, additional studies are needed to determine the mechanisms of H2Aub propagation in the context of natural recruitment and how its spreading is orchestrated by PRC1 and PR-DUB/BAP1. Clearly, future work is needed to better position H2Aub in the epigenomic landscape and determine how deregulation of this modification underlies human disease.
